# Why educators endorse a neuromyth: relationships among educational priorities, beliefs about learning styles, and instructional decisions

**DOI:** 10.3389/fpsyg.2024.1407518

**Published:** 2024-06-17

**Authors:** Christine Bresnahan, Emily Grossnickle Peterson, Courtney Hattan

**Affiliations:** ^1^Department of Neuroscience, American University, Washington, DC, United States; ^2^School of Education, American University, Washington, DC, United States; ^3^School of Education, University of North Carolina at Chapel Hill, Chapel Hill, NC, United States

**Keywords:** neuromyths, learning styles (LS), meshing hypothesis, multimodality, instructional design

## Abstract

Despite evidence to the contrary, many people believe in learning styles (LS)–the idea that students learn best in their preferred modality, such as visual, auditory, or kinesthetic. However, the impact of this belief on instructional decisions remains unclear. Therefore, this study investigated how belief in the neuromyth impacts instructional choices and why educators choose an LS lesson plan or an alternative. We found that educators’ beliefs about LS indeed predicted their instructional choice, but that other factors influenced their decisions as well. Three themes encapsulate educators’ justifications for their lesson plan choices: beliefs about LS, practical considerations, and student learning and motivation. These findings suggest that for many educators, implementing an LS lesson provides an opportunity to integrate diverse teaching strategies that address multiple educational priorities. Although many prior studies have replicated the prevalence of the myth, this is one of the first to explore the reasons that LS is attractive to educators. Attempts to dispel the LS neuromyth could leverage the reasons educators find LS appealing to provide alternative research-backed approaches to meet their goals. Future research should examine the extent to which beliefs in the LS neuromyth are translated into instructional practices within classroom lessons and explore potential differences across grade levels.

## Introduction

Neuromyths are widely held misinterpretations of cognitive or neuroscience research (Grospietsch and Lins, 2021). Unsurprisingly, many of the neuromyths that pertain to thinking and information processing have flowed into education, as teachers actively seek new research on learning and the brain (Dekker et al., 2012; Macdonald et al., 2017). One such neuromyth involves the concept of modality-specific learning styles (LS), such as visual, auditory, and kinesthetic (sometimes referred to as VARK, which includes a reading/writing learning style). The LS neuromyth states that each individual has a learning style, based on their preference, and will learn better in that modality (Dinsmore et al., 2022).

### Learning styles neuromyth

Ubiquitous and persistent, the LS neuromyth may have emerged from the fact that people express opinions about the modality in which they prefer to learn information (Grospietsch and Lins, 2021). In a logical extension, many assume that a preferred modality for receiving information is the best modality for learning information. However, this neuromyth misconstrues scientific findings in a few fallacious ways. First, one predominant misinterpretation centers around the assumption that learning styles are inherent characteristics and that people will learn best when instructed in their preferred LS (Grospietsch and Lins, 2021; Dinsmore et al., 2022). The learning style neuromyth assumes *a priori* that intellectual styles of learning—defined by sensory input channels—exist, and that they are static aspects of learners (Pasquinelli, 2012). Furthermore, many people incorrectly assume that a learning style can be identified for students, such as through specialized LS assessments, so that the teachers can deliver instruction in accordance with the test results (e.g., Grospietsch and Lins, 2021).

A second common misconception is that people learn better when they receive information in their preferred LS, known as the meshing hypothesis (Pashler et al., 2008). This is not true. While people commonly report preferring to learn in a certain modality, aligning instruction to the preferred LS does not benefit the learner (Pashler et al., 2008; Riener and Willingham, 2010; Rogowsky et al., 2015; Newton and Salvi, 2020).

Despite the lack of evidence for each of these assumptions, belief in the learning style neuromyth is prevalent and persistent. Although educators demonstrate greater ability to recognize neuromyths compared to the general public, they still maintain a high level of endorsement for these misconceptions (e.g., Macdonald et al., 2017; Hattan et al., 2024). According to prior studies, over 75% of educators agree with the claim that students learn best in their preferred learning style (Macdonald et al., 2017; Hughes et al., 2020; Newton and Salvi, 2020). This high rate has persisted, even among those with high neuroscience knowledge, despite decades worth of research showing that adhering to the learning style is ineffective for supporting learning (Macdonald et al., 2017; Newton and Salvi, 2020). Additionally, Hughes et al. (2020) found that belief in neuromyths decreases with additional formal teacher training, but increases with neuroscience exposure through other venues.

Belief in LS remains a highly pertinent research topic in part because many authors posit that its persistence in educational settings may have pernicious consequences. Labeling students with their LS perpetuates fixed mindsets and decreases student flexibility and motivation (Papadatou-Pastou et al., 2021). Acceptance of LS may pigeonhole students’ current activities or future career paths (Riener and Willingham, 2010). Moreover, belief in an incorrect teaching method leads to time, effort, and money spent on an ineffective practice instead of a beneficial one (Dekker et al., 2012; Macdonald et al., 2017). Other authors assert that belief in the LS neuromyth does not impact teacher quality (Horvath et al., 2018; Krammer et al., 2021). For instance, a study looked at academic achievement of first year education majors and found that there was no difference in grades based on beliefs in neuromyths (Krammer et al., 2021).

### The present study

Although many studies have shown a high proportion of educators believe the LS neuromyth to be true (Horvath et al., 2018; Krammer et al., 2021), little is known about why educators endorse this idea or how the persistence of the neuromyth impacts instruction. To decrease belief in an ineffective teaching practice, we need to first understand how and why educators are using it in the classroom. Therefore, our study investigated how the belief in LS impacted educators’ anticipated instructional decisions and why educators made those choices.

Specifically, we investigated the following research questions:

*RQ1*. Do educators’ stated beliefs about LS predict whether they chose an LS lesson plan?

*RQ2*. What justifications do educators provide for choosing an LS lesson or an alternative option?

## Methods

### Participants

Sixty current U.S. educators participated in the study. Informed consent was obtained from each participant before the study. Participants were recruited via Prolific.co and were compensated $1.28 ($9.60/h) upon completion of the study. Participants were only invited to complete the survey if they indicated on Prolific’s prescreening survey that their current job is in the education sector and involves teaching. Given that this was a preliminary investigation of educators’ reasons for endorsing the LS neuromyth, we felt it was important to include a diverse range of teaching roles across grade levels, subject areas, and locations within the U.S. Three participants failed the attention check on the questionnaire and were excluded from the quantitative analysis of that measure. Sociodemographic characteristics of participants are included in Table 1.

**Table 1:** Sociodemographic characteristics of participants.

Baseline characteristic	*n*	%
Race/ethnicity/ethnicities		
White	53	88%
Black or African American	4	7%
American Indian or Alaska Native	1	2%
Asian	5	8%
Latino/Latina/Latinx Hispanic	1	2%
Middle Eastern/North African (MENA)	1	2%
Highest degree		
Teaching license outside of degree program	2	3%
Associate’s degree/2 year college degree	2	3%
Bachelor’s degree/4 year college degree	16	26%
Master’s degree	30	50%
PhD or EdD	9	15%
JD	1	2%
Primary job title		
Teacher	26	43%
Teaching assistant	9	15%
K-12 administrator	1	2%
Professor	18	30%
Substitute teacher	3	5%
Other^a^	3	5%
Grades taught (for more than 3 months)		
Pre-K	12	20%
Elementary (K-5)	25	42%
Middle school (6–8)	21	35%
High school (9–12)	25	42%
Other^b^	22	37%

*N* = 60. ^a^School psychologist, college level instructor, education therapist. ^b^Undergraduate and graduate level courses.

### Procedure

First, participants read a hypothetical teaching scenario, chose between recommending the use of an LS-based lesson or a multimodal lesson, and explained their choice. Second, participants rated their agreement with statements about LS. Participants then responded to a brief demographics questionnaire. The scenario measure was presented first to solicit answers that were not influenced by the questions in the LS questionnaire measure.

### Measures

#### Teaching scenarios

In the scenarios measure, participants read one of three lesson scenarios, randomly assigned, each of which briefly described a grade level and lesson goal. After reading the scenario, participants chose one of two instructional designs to teach the lesson. The order in which the two choices were presented to participants was randomized.

The choices were written so that one depicted a lesson using LS and the other depicted a lesson using multiple modalities. In the LS option, each student would receive the lesson in only one of the modalities according to whether they were an auditory, visual, or kinesthetic learner. This design would enable each student to learn the topic in one LS. In the multimodality option, the lesson was presented in all three of the modalities sequentially. This design would enable all students to learn the topic in multiple modalities. Full text for all scenarios and choices is included in Supplementary Information.

After reading the scenario and selecting their preferred instructional design, participants justified their choice by writing “one paragraph that justifies your choice for the scenario above.” They typed their responses in an open-ended answer box that had a character minimum of 100 and no character maximum.

#### Learning styles questionnaire

The questionnaire measure consisted of a five-item survey about LS (see Supplementary Information). Participants rated the strength of their agreement with statements about LS using a six-point scale from *Strongly Disagree* to *Strongly Agree*. Their explicit belief in the LS theory was measured with the item, “Individuals learn better when they receive information in their preferred learning style (e.g., auditory, visual, and kinesthetic),” developed by Dekker et al. (2012). Their views on the meshing hypothesis were also measured for each modality (e.g., “It is important for teachers to match auditory learners to auditory content”). Scores on the five items were averaged. Scale reliability was high (*ɑ* = 0.97).

### Data analysis

#### Quantitative analysis

To examine whether educators’ beliefs about learning styles predicted whether they endorsed an LS approach to the teaching scenario (RQ1), we regressed scenario choice (1 = selection of the LS option) on the LS questionnaire controlling for participants educational background (1 = graduate degree in education, 0 = no graduate degree in education). Data analyses were conducted in Stata 16 (StataCorp, 2019) and RStudio (RStudio Team, 2020).

#### Qualitative analysis

Open-ended responses to the Teaching Scenarios measure were coded and qualitatively analyzed. Our qualitative analysis consisted of two cycles, each with successive rounds of coding, categorizing, discussion, and agreement.

The first coding cycle utilized three main coding methods, conducted in rounds. First, we coded all responses with Initial Coding, which segments and examines responses for commonalities and differences (Saldaña, 2016). Most responses provided multiple reasons for their choices, so each response was broken into one or more idea units corresponding to each reason. Second, we coded the idea units that referred to LS or multimodality with Values Coding, which identifies the values, attitudes, and beliefs of responses (Saldaña, 2016). Third, we employed Descriptive Coding (Saldaña, 2016) to identify the topics in all idea units unrelated to modality. Following code development, all authors reviewed and discussed the codes and the fit of the examples.

Upon agreement, we conducted the second coding cycle, to identify and define broader categories and themes, based on the codes that were identified. In this cycle, we categorized codes, refined the coding categories, and discussed the fit of codes. For each category, we created a detailed description, inclusion and exclusion criteria, lists of coded idea units that fit the category, and lists of coded idea unit non-examples. From the codes and categories, we identified three themes: beliefs about modalities, practical considerations, and student learning and motivation. The latter two themes required an additional round of peer-debriefing to develop fully.

## Results

The data are available at https://osf.io/4r9n3/ (anonymized for peer review).

### Quantitative results

Consistent with prior research (Macdonald et al., 2017; Hughes et al., 2020), most participants agreed that matching instruction to a student’s learning style is beneficial for learning. Specifically, 82.5% (*n* = 47) of educators’ questionnaires indicated agreement (*somewhat agree, agree*, or *strongly agree*), with an average rating between *somewhat agree* and *agree*, *M* = 4.44, *SD* = 1.03 (see Supplementary Information). However, slightly fewer than half of educators (45.6%) endorsed the LS scenario lesson option. See Table 2 for a summary of questionnaire scores by teaching scenario choice.

**Table 2:** Summary of learning styles scenario choices and learning styles questionnaire.

Teaching scenario choice	Scenario selection		Learning styles questionnaire average	
	*N*	%	*M*	*SD*
Learning styles option	26	45.61	4.97	0.74
Multimodal option	31	54.39	3.99	1.03
Total	57	100	4.44	1.03

Scores on the LS questionnaire predicted scenario choice, *χ*^2^(1, *N* = 57) = 15.73, *p* = 0.0001, Pseudo *R*^2^ = 0.20. This relation held even after controlling for participants’ educational background, *χ*^2^(2, *N* = 57) = 15.80, *p* = 0.0004. Specifically, the odds of selecting the LS option for the scenario was four times greater for each one point increase on the LS questionnaire, *OR* = 3.99, 95% CI [1.69, 9.52].

### Qualitative results

We identified three major themes in educators’ reasons for choosing one of the lesson scenarios: beliefs about learning modalities, practical considerations, and student learning and motivation. Over half of the participating educators (76.7%, *n* = 46) expressed modality beliefs when justifying their lesson plan choice. A fifth of the educators (20.0%, *n* = 12) prioritized practical considerations justifying their lesson plan choices. Under half of the educators (42.0%, *n* = 25) considered student learning and motivation when explaining their lesson plan choice. We highlight findings from each of the three themes, with additional examples provided in Table 3.

**Table 3:** Sample quotes and frequencies for participant responses in each theme.

Category	Sample quote	Scenario choice	*n*	%
Theme 1: Beliefs about learning modalities				
Embraces LS	I like the idea of the teacher being able to meet the needs of different students and their learning styles.	LS	26	43.3
Rejects LS	Learning styles are not a scientific thing. There is no evidence to back up that some people are “auditory learners” or “visual learners” etc.	MM	16	26.7
Embraces MM	…What is best for students is a mix of presentation styles to engage multiple parts of the brain.	MM	16	23.3
Total			46	76.7
Theme 2: Practical Considerations				
Logistical constraints	There are a number of reasons I chose this option: (1) less work for the teacher. (2) The teacher and students do not know exactly what kind of learner they are. (3) Everyone can benefit from the various learning styles. It gives all the students a more well-rounded idea of what they are learning.	MM	9	15.0
Planning time limits	I think this one is the best because it will require less planning on the part of the teacher…	MM	4	6.7
Teaching time limits	…This will be the most successful based on how much time you have to teach this section…	MM	5	8.3
Classroom management	It’ll be too difficult to oversee three different groups of children at the same time, much less assess and evaluate them…	MM	6	10.0
Total			12	20.0
Theme 3: Learning and Motivation				
Motivational Factors	Inclusivity is important with learning. There is not one right way to teach a topic and teachers must provide varying methods. A student can have more than one learning style.	MM	19	31.7
Choice	Students have the option of choosing their approach to the subject. [It] allows for a greater degree of individual learning preferences.	LS	7	11.7
Engagement	…Additionally, [the LS lesson] creates engagement because students will be more motivated to do the activity that is most relevant for them.	LS	6	10.0
Learning community	…Also, the students would be able to interact and learn from each other as a whole group and not [be] compartmentalized.	MM	7	11.7
Teaching practices	The [MM] option does not allow for a whole lot of student-led exploration, but allows different ways of engaging with the content and has both scaffolds and forms of assessment. The [LS] option differentiates but not in a way supported by educational theory, and two groups do not have a stated form of assessment. There is no mention of scaffolding or supports in the [LS option].	MM	13	21.7
Differentiation	[The LS lesson] differentiates for various student needs and allows students to succeed in their own way. Student X might need a different way of understanding than Student Y so this method allows them all to achieve their highest potential.	LS	4	6.7
Scaffolds	I like the use of scaffolds in the first scenario…	MM	4	6.7
Assessment	… [The LS lesson] is diversified according to the student’s preferred way of learning. It assesses them based on those ways of learning which makes it more fair to each student.	LS	7	11.7
Total			27	45.0

Categories are not mutually exclusive, therefore percentages do not add to 100%. LS, learning styles; MM, multimodality.

#### Theme 1: beliefs about learning modalities

The majority of participants included beliefs about modalities as justifications for selecting a lesson option. These responses fit into three categories: embraced LS, rejected LS, or embraced multimodality.

In the first category, participants endorsed the LS neuromyth directly, agreeing that (a) students have preferred LS, and (b) students learn best in that style. For instance, one substitute teacher wrote, “I think teaching by learning style will help ensure all the students have the best chance at fully understanding the lesson.” Other responses that embraced LS referred to aspects that educators inferred were inherent in LS, for instance, differentiated instruction. A college-level teaching assistant justified their selection noting, “This style [lesson plan] seems better because it caters to different learners. This seems like it is a more measured approach.” Thus, of those who spoke positively of LS, not all expressed agreement with the LS neuromyth in its entirety.

The second category in this theme included responses that expressed a belief that rejected LS. Some explicitly debunked LS, providing justifications such as, “The learning styles method where particular learners use their preferred learning style has been debunked. Most students benefit from multiple modalities.” Others cited specific reasons for eschewing LS, such as one educator, with experience teaching grades from PreK through high school, who said, “I’m not sure that visual/auditory/kinesthetic learning thing is something you should tailor your instruction toward. Everyone needs to develop all these.” A college professor justified their choice of multimodal lesson, explaining, “I think it is important to introduce to the whole class first, as a group…” Meanwhile, they articulated mixed feelings about LS, adding that, “…While I like individual learning styles, I thought that was based on old data.”

The third category of Theme 1 responses included participants who embraced the concept of multimodal lessons. For example, an elementary school psychologist noted in their response that, “[Students] benefit from visual and auditory learning, therefore, the [MM] option would be more effective.” Some of the responses utilized language associated with the LS neuromyth despite rejecting the principles of LS. For example, a professor with prior experience teaching in primary and secondary education explained, “The second option does not work that well as it forces certain learners to do certain things, and learning styles should be blended together for a more inclusive and powerful lesson….”

**Figure 1:**
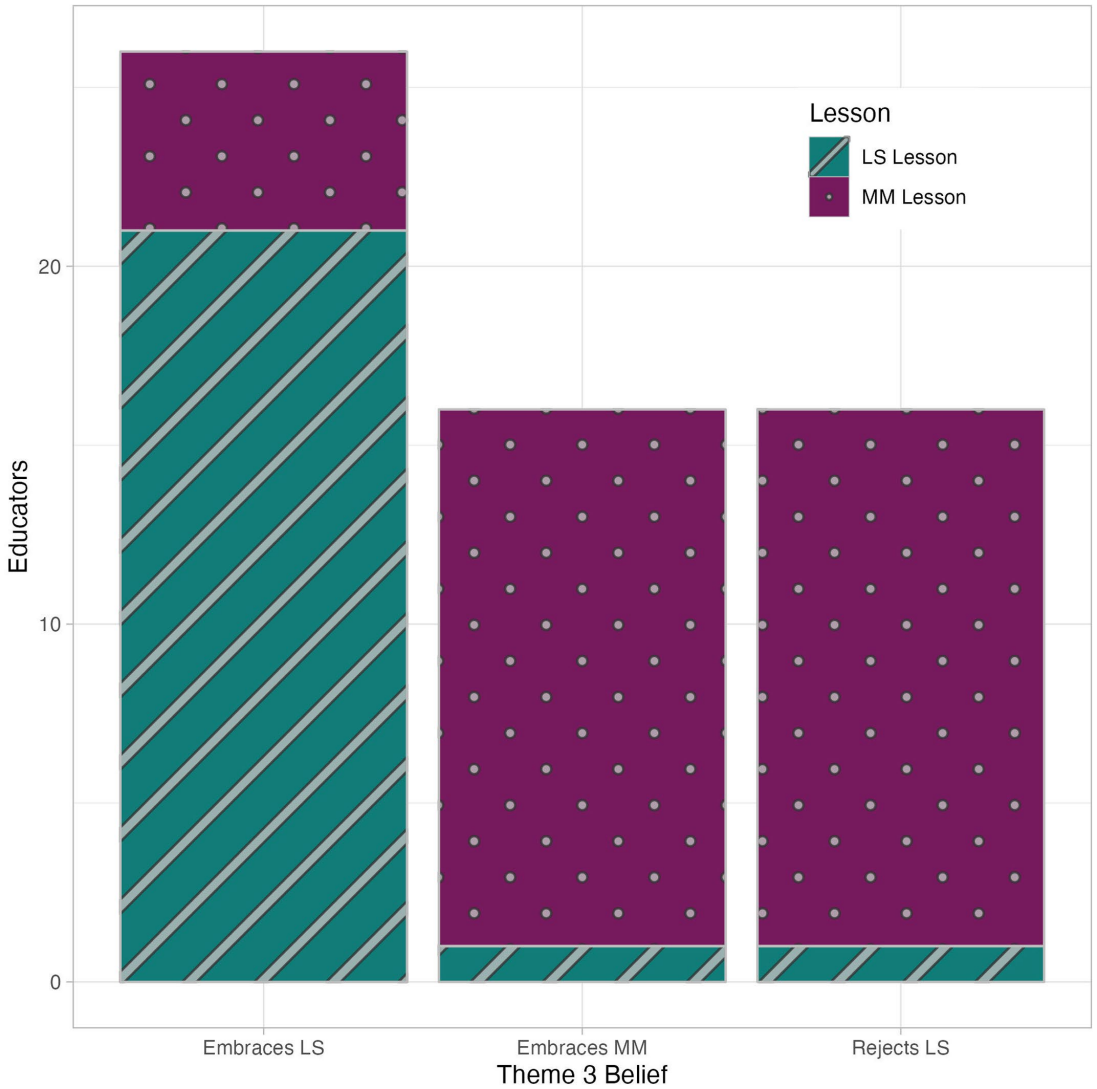
Mild mismatch between educators’ beliefs about LS and lesson plan choices. LS, learning styles; MM, multimodality.

Finally, while most educators’ justifications aligned with their scenario choice, some justifications did not match (see Figure 1). For example, a college professor who chose the LS option stated, “I do not think either design is really [optimal] because learning styles have been debunked in numerous studies. I think all students should be exposed to each of the different methods of learning the content and should either be given an option of how to be assessed or be given multiple assessment methods. Students need to learn to adapt to multiple ways of learning.”

#### Theme 2: practical considerations

The second theme included responses that justified their lesson plan choice with practical considerations, in the categories of logistical constraints (e.g., planning time) and classroom management considerations. Notably, despite recognizing potential problems with one of the lesson plans, certain educators opted for that one regardless, due to other priorities. For example, a high school teacher chose the LS lesson, but acknowledged that the MM lesson, “…may be appropriate based on class size, time, resources, etc.” All of the educators who noted a practical consideration, but made their choice based on other factors, chose the LS lesson, while all of the educators who justified their choice with practical considerations selected the multimodality lesson.

Several participants’ explanations fell within the category of logistical constraints: restrictions on resources in preparing or delivering lessons, including limitations on planning time. A teaching assistant with experience in middle school grades chose the multimodality option and explained, “…in a real-world classroom setting the ability to split a middle school class into three groups, and have three different lessons planned, is not often realistic.” Time constraints for teaching also influenced educators’ decisions, including a high school teacher who noted, “Within the time constraints, option two [MM] is the most likely to get the information across in a meaningful way.”

A handful of participants noted concerns about a second category—classroom management—especially regarding the three different activities in the LS lesson. For example, a college professor rejected the LS lesson because, “Whenever you separate students into groups, it can be problematic.” Another professor explained that they chose the MM lesson, because it would be easier to, “…keep kids on task to do each of the three different types of learning…” and that for first grade students, “…Releasing them to various stations sounds chaotic to me.”

#### Theme 3: student learning and motivation

Theme 3 included the responses of 27 educators (45%) who justified their instructional decision based on two categories: (1) motivational factors, which included elements that increase students’ desires to move toward learning or performance goals (Covington, 2000), or (2) teaching practices: pedagogical strategies that impact lesson plan design.

Responses within the motivational factors category were roughly divided evenly among three sub-categories of choice, engagement, and fostering a positive learning community. Several educators explicitly mentioned providing students with choice, which was unexpected, as neither lesson plan mentioned choice. In fact, although researchers typically interpret teaching based on LS as assigning modality-specific activities based on a students’ *a priori* determined style, multiple educators inferred that students would be given a choice among the activities. An educator who chose the LS lesson stated, “…Giving students the ability to choose how they learn will likely increase engagement and help students make meaningful connections and memories…” The interpretation of choice also showed up in justifications for the MM lesson, such as one educator who explained, “…having the choice to move between multiple ones can help them become strong in all areas.” Engagement, often conceptualized as a person’s interest propelling them to actively orient toward and participate in their current task (Bryson and Hand, 2007; Bailey et al., 2015), was also important to educators’ decisions. One high school teacher directly connected choice, engagement, and motivation in their response; “…Additionally, [choice] creates engagement because students will be more motivated to do the activity that is most relevant for them.” Finally, some educators mentioned elements that impact classroom community and student socio-emotional well-being, such as increasing inclusivity, learning from peers, and decreasing self-consciousness. The justifications of two educators, at either end of the grade range, show the variety of codes within this category. A teacher at the pre-K level explained, “…Also, when the students do the acting out activity, they can all benefit from working together…” A professor teaching higher education courses rejected the LS lesson plan because, “…Students may feel inferior to their peers by being tracked into different activities. The option I chose presents information in a variety of ways without dividing up the class.”

Other educators focused on the impact of teaching practices on student learning and motivation, such as differentiation, scaffolding, and assessment. A few educators noted that the LS lesson provided differentiated instruction because they interpreted modality as a means of differentiation. For instance, an educator with experience in classrooms in elementary and high school, wrote, “[The LS lesson] appeals to different learning styles for differentiated instruction. Since students learn differently it’s appropriate to try to change the way the lesson is presented to the students in the classroom so that they are better able to learn the materials.” In contrast, an elementary educator who identified differentiation in the LS lesson but chose the MM lesson explained, “…The [LS] option differentiates but not in a way supported by educational theory…” Finally, several educators referred to scaffolds or assessments in their responses. One educator at the university level supported their choice, saying, “I like the use of scaffolds in the [MM] scenario….”

## Discussion and implications

The present study investigated how beliefs about LS impacted educators’ instructional decisions and why educators opted to choose or reject a lesson plan that incorporated LS. We found that approximately 82% of educators believed in LS, replicating prior research (79% in Hughes et al., 2020; 76% in Macdonald et al., 2017; 89% in Newton and Salvi, 2020). Furthermore, the strength of teachers’ beliefs about LS predicted their instructional decisions. We utilized qualitative analysis to explore the justifications that educators provided for their instructional decisions, and found three themes: beliefs about learning modalities, practical considerations, and student learning and motivation. Through the use of a novel scenario-based measure and educators’ justifications for their instructional decisions, our findings help unpack what draws educators toward (and away from) the implementation of LS during classroom lessons.

### Educators perceive LS as a vehicle for choice and differentiation

First, educators appreciated ancillary benefits that they perceived in LS. Although many of the educators who selected the LS lesson did so because they believed that matching LS to instruction was best, other educators who chose the LS lesson did so because they saw it as an opportunity to incorporate other teaching techniques, such as choice and differentiated instruction. For example, educators perceived the LS lesson as a means of recognizing and supporting individual differences, as a type of differentiation. If the goal is to decrease educators’ beliefs about LS, attempts to dispel this myth need to provide compelling alternatives that enable educators to implement strategies such as choice and differentiation that will support their students.

### Educators have nuanced interpretations of LS compared to researchers

Second, research studies often strictly define LS according to the meshing hypothesis, utilizing the Dekker et al. (2012) questionnaire item, *“*Individuals learn better when they receive information in their preferred learning style.” In contrast, we found that some educators define LS more loosely. For example, some educators expressed beliefs that sensory modes exist, without embracing the meshing hypothesis. One referred to “overlapping learning styles” and another opined that, “… multiple [LS] can help [students] become strong in all areas.” Though agreeing that students learn best in one modality in the questionnaire and using the corresponding phrase “learning styles” in their answers, these responses indicate a more nuanced view of learning modalities. This variability and the lack of consensus among educators on the definition of LS may skew interpretations of quantitative surveys on beliefs about LS. However, it also provides important insight: educators and researchers may not hold the same understanding of what it looks like to implement LS in the classroom.

### Educators believe LS lessons involve costs in preparation time and classroom management

Finally, educators’ justifications show conflicting priorities. A few educators who chose the LS lesson identified a drawback to LS, such as, “…Sure, [LS] takes more planning, but…,” and then explained why the benefits outweigh the costs. In contrast, several educators rejected the LS lesson due to such obstacles. Their responses suggest that an LS lesson requires more effort—in preparation time and classroom management—according to both educators who chose the MM lesson and educators who chose the LS lesson. This evidence lends credence to prior authors’ trepidation that belief in LS may lead to wasted time and effort for educators (Dekker et al., 2012; Macdonald et al., 2017). It also suggests that appealing to educators’ need for efficient and effective use of planning and instructional time may help to make the case against using LS.

### Implications for educational practice

These results have important implications for curriculum design and teacher education. Unfortunately, teacher education curricula continue endorsing the LS neuromyth (Blanchette Sarrasin et al., 2019; Den Dekker and Kim, 2022). Especially given our finding that beliefs in the LS neuromyth predict instructional decisions, teacher education curricula should work to dispel this myth and instead emphasize practices such as choice and differentiation. Our findings suggest that, although teacher belief in the LS neuromyth remains high, the reasons and ways in which educators incorporate it into their classrooms are diverse. Recent research has suggested that relatively short interventions can decrease educator belief in the LS neuromyth (Hattan et al., 2024). When attempting to debunk this neuromyth, it is important to first validate potential benefits of such a concept, such as choice and differentiation, and then the incorrect components of LS need to be refuted. The final step is to present alternative ways to obtain those benefits more efficiently using research-backed practices.

### Limitations and future directions

The present study sheds light on the influence of belief in LS on instructional decisions and provides insight into why educators choose an LS or alternative lesson; however, there are several limitations that should be acknowledged. Although we attempted to create parallel scenarios, a couple discrepancies emerged in the attempt to make the two scenarios distinct; only the MM lesson plan description specified scaffolds and only the LS lesson plan described an assessment. Despite these differences, scaffolds and assessments only represented a small portion of the reasons teachers provided for their lesson choice. Future research should examine whether certain features of lesson plans make them more or less likely to be adopted and whether teachers believe those features influence the implementation of multi-modal lessons relative to LS lessons. Moreover, it would be ideal to observe actual lessons rather than analyzing their anticipated choices based on hypothetical scenarios. Regardless, the justifications provided more information than that obtained from self-report questionnaires used in prior research on LS beliefs. Finally, it is important to note that the relatively small sample in the present study was conducted with predominantly White teachers in the U.S across a range of educator roles and levels. Furthermore, the scenarios all described hypothetical teaching scenarios in elementary and middle school, which not all participants may have had experience teaching. Future research should be conducted with larger samples and examine the extent to which these findings generalize across educators, including the degree to which factors such as grade level matter.

Overall, the results of this study provide valuable insights into the impact of belief in LS on instructional decisions and contribute to our understanding of why educators chose the LS or alternative lesson. The majority of teachers made instructional decisions based on a desire to support students’ success, and we hope future research explores how to leverage teachers’ desires to do so most effectively.

## Data availability statement

The datasets presented in this study can be found in an online repository accessible via the following link: https://osf.io/4r9n3/.

## Ethics statement

The studies involving humans were approved by American University Institutional Review Board, Office of Research Integrity. The studies were conducted in accordance with the local legislation and institutional requirements. The participants provided their written informed consent to participate in this study.

## Author contributions

CB: Writing – review & editing, Writing – original draft, Visualization, Methodology, Formal analysis, Data curation. EP: Conceptualization, Formal analysis, Investigation, Methodology, Project administration, Supervision, Writing – review & editing. CH: Writing – review & editing, Project administration, Methodology, Investigation, Conceptualization.
