# Supplementary material for: Why educators endorse a neuromyth: relationships among educational priorities, beliefs about learning styles, and instructional decisions
Source: Front Psychol. 2024 Jun 17;15:1407518. doi: 10.3389/fpsyg.2024.1407518 (PMC11215186; doi:10.3389/fpsyg.2024.1407518)
Supplement: Supplementary file 1 [file Data_Sheet_1.docx]

# SUPPLEMENTARY MATERIAL

## Full Scenarios Texts

In this study you will be asked to answer some questions about strategies for teaching and learning. Please respond based on your knowledge and experiences as a teacher.

### Scenario 1

Please read the scenario. Then, select one of the two options that you think is the best instructional decision and justify your choice.

A 4th grade teacher is planning a science lesson about ecosystems. The goal of the lesson is for students to be able to explain the interdependence between the various animals who live in a desert ecosystem.

In order to meet the learning needs of all students, which one of the following two options would be the best instructional decision?

Option A

The teacher will develop three different activities according to students’ learning styles. Auditory learners will listen to the teacher describe a desert ecosystem, visual learners will read a text and see a labeled diagram of the ecosystem, and kinesthetic learners will create a diorama of a desert ecosystem. Students will receive instruction according to their preferred learning style and will also be assessed based on their learning style.

Option B

The teacher will introduce a diagram of a desert ecosystem to the whole class. Then, the teacher will read aloud a text and support students as they complete a graphic organizer. Finally, students will create a diorama depicting how different animals depend on each other. Instructional scaffolds such as vocabulary supports and partially completed graphic organizers will be provided.

Please justify your choice for the scenario above.

### Scenario 2

Please read the scenario. Then, select one of the two options that you think is the best instructional decision and justify your choice.

A 7th grade teacher is planning a history lesson about the Great Migration. The goal of the lesson is for students to be able to explain why many Black Americans moved from the south to the north after World War I.

In order to meet the learning needs of all students, which of the following two options would be the best instructional decision?

Option A

The teacher will develop three different activities according to students’ learning styles. Auditory learners will listen to the teacher describe the Great Migration, visual learners will read a text and view an interactive map that illustrates the migration, and kinesthetic learners will participate in a gallery walk that includes multiple texts and pictures documenting the Great Migration. Students will receive instruction according to their preferred learning style and will also be assessed based on their learning style.

Option B

The teacher will introduce an interactive map that illustrates the Great Migration to the whole class. Then, students will participate in a gallery walk where they will take notes via a graphic organizer as they view multiple texts and pictures documenting the Great Migration. Finally, students will write a paragraph describing why many Black Americans participated in the Great Migration. Instructional scaffolds such as vocabulary supports and partially completed graphic organizers will be provided.

Please justify your choice for the scenario above.

### Scenario 3

Please read the scenario. Then, select one of the two options that you think is the best instructional decision and justify your choice.

A 1st grade teacher is planning a social studies lesson on the various roles that people can have within a community. The goal of the lesson is for students to be able to explain how all people, not just official leaders, are important members of our society.

In order to meet the learning needs of all students, which of the following two options would be the best instructional decision?

Option A

The teacher will introduce pictures that depict varying community members to the whole class. Then, students will listen as the teacher reads aloud a book about different community roles. Finally, students will act out what it means to be a teacher, scientist, mayor, mail carrier, doctor, cook, or other roles they can think of. Instructional scaffolds such as vocabulary supports will be provided.

Option B

The teacher will develop three different activities according to students’ learning styles. Auditory learners will listen to the teacher read a book about varying community roles, visual learners will look at pictures that depict community members completing different tasks, and kinesthetic learners will act out what it means to be a teacher, scientist, mayor, mail carrier, doctor, cook or other roles. Students will receive instruction according to their preferred learning style and will also be assessed based on their learning style.

Please justify your choice for the scenario above.

## Level of Agreement and Prevalence of Learning Styles Beliefs

|  | Level of Agreement^a^ | | | | | | Prevalence of LS Belief |
| --- | --- | --- | --- | --- | --- | --- | --- |
| Neuromyth | 1 | 2 | 3 | 4 | 5 | 6 | (4–6) |
| Individuals learn better when they receive information in their preferred learning style (e.g., auditory, visual, kinesthetic). | 2% | 5% | 4% | 35% | 35% | 19% | 89% |
| It is important for teachers to match auditory learners to auditory content. | 2% | 7% | 9% | 39% | 35% | 9% | 82% |
| It is important for teachers to match visual learners to visual content. | 2% | 7% | 9% | 35% | 37% | 11% | 82% |
| It is important for teachers to match kinesthetic learners to kinesthetic content. | 2% | 7% | 11% | 33% | 35% | 12% | 81% |
| Some students learn best by looking at graphics, while other students learn best by working with their hands. | 0% | 4% | 4% | 28% | 39% | 26% | 93% |
| *Note*. LS = Learning Styles . ^a^(1) Strongly disagree, (2) Disagree, (3) Somewhat disagree, (4) Somewhat agree, (5) Agree, and (6) Strongly agree. | | | | | | | |
